# Novel Two-Dimensional Conjugated Polymer Containing Fluorinated Bithiophene as Donor and Benzoselenodiazole as Acceptor Units with Vinyl-Terthiophene Pendants for Polymer Photovoltaic Cells

**DOI:** 10.3390/polym9070272

**Published:** 2017-07-07

**Authors:** Rathinam Raja, Shengkai Luo, Chuen-Yo Hsiow, Syang-Peng Rwei, Leeyih Wang

**Affiliations:** 1 Center for Condensed Matter Sciences, National Taiwan University, Taipei 10617, Taiwan; t101320391@ntut.org.tw (S.L.); chuenyohsiow@ntu.edu.tw (C.-Y.H.); 2 Institute of Organic and Polymeric Materials, National Taipei University of Technology, Taipei 10608, Taiwan; f10714@ntut.edu.tw; 3 Institute of Polymer Science and Engineering, National Taiwan University, Taipei 10617, Taiwan

**Keywords:** benzoselenodiazole, vinyl-terthiophene, difluoro-bithiophene, polymer solar cell

## Abstract

Novel two-dimensional conjugated copolymer, abbreviated as PDTBSeVTT-2TF, containing electron-deficient 4,7-di(thiophen-2-yl)benzo[*c*][1,2,5]selenodiazole (DTBSe) unit, conjugated vinyl-terthiophene (VTT) side chain and 3,3′-difluoro-2,2′-bithiophene (2TF) was designed and synthesized using microwave-assisted Stille cross-coupling polymerization. UV–visible absorption and cyclic voltammetry studies revealed that this copolymer possesses a strong and broad absorption in the range of 300–800 nm and a narrow optical bandgap (*E_g_*) of 1.57 eV with low-lying HOMO and LUMO energy levels. Further, the bulk heterojunction polymer solar cells (PSCs) were fabricated using PDTBSeVTT-2TF as donor and [6,6]-phenyl-C_71_-butyric acid methyl ester (PC_71_BM) as acceptor with an inverted device structure of ITO/ZnO/PDTBSeVTT-2TF:PC_71_BM/V_2_O_5_/Ag. The processing temperature of blend solution for preparing PDTBSeVTT-2TF:PC_71_BM active layer showed obvious impact on the photovoltaic performance of solar devices. The cell fabricated from the blend solution at 65 °C exhibited enhanced power conversion efficiencies (PCE) of 5.11% with a *J_sc_* of 10.99 mA/cm^−2^ compared with the one at 50 °C, which had a PCE of 4.69% with a *J_sc_* of 10.10 mA/cm^−2^. This enhancement is due to the dissolution of PDTBSeVTT-2TF clusters into single molecules and small aggregates, improving the miscibility between the polymer and PC_71_BM and thus increasing the donor/acceptor interface.

## 1. Introduction

Thin-film bulk heterojunction (BHJ) polymer solar cells (PSCs) composed of conjugated polymer as electron donor (D) and fullerene derivatives as electron acceptor (A) are promising for harvesting unlimited renewable energy from the sun [1,2,3,4]. These PSCs were highly in demand for their potential cost-effective option in current photovoltaic technology owing to their several advantages including light weight, mechanical flexibility, and solution processability [5,6]. Recently, the power conversion efficiency (PCE) of PSCs has been dramatically increased up to 12% [7,8,9,10,11], through novel design of low bandgap copolymers with suitable HOMO and LUMO energy levels and optimization of processing condition, device structure and morphology. In addition, significant developments in device performance have been accomplished by sustainable atom-economical synthetic methodologies [12]. In spite of this impressive PCE of PSCs, a further improvement in cell performance and operational lifetime is still needed for commercialization. The key factors that enhance the efficiency of PSC include open-circuit voltage (*V_oc_*), short-circuit current density (*J_sc_*), and fill factor (FF). An ideal polymer donor should have broad absorption in near infrared (IR) region and narrow optical bandgap to ensure the harvest of more solar photos to generate larger short circuit-current (*J_sc_*), a deep HOMO energy level to improve the material’s stability and result in a high *V_oc_* device, and good hole mobility to obtain high FF [13,14].

Recent advances in designing and synthesizing narrow bandgap polymers are based on alternating donor–acceptor copolymer containing an electron-rich (donor) and an electron-deficient (acceptor) moieties into the polyconjugated backbone and this combination is considered as one of the successful strategies in reducing the bandgap and decreasing the HOMO levels, thereby allowing intermolecular charge transfer and thus leading to enhanced photovoltaic performance [15,16]. Recently, the development of benzodithiophene/thieno[3,4-*b*]thiophene-based low-bandgab polymers such as PTB7 and PTB7-Th and device engineering significantly increased the PCE up to 11% [17,18]. In addition, several research groups incorporated electron-withdrawing fluorine atoms into the conjugated polymer backbone to minimize steric interactions and lower the HOMO and LUMO energy levels [19]. Furthermore, the fluorine atoms on the polymer backbone strongly induced dipole interactions with hydrogen or sulfur atoms (F…H or F…S) that can promote molecular co-planarity and intra/inter-molecular interactions, resulting in enhanced charge mobility and FF of the polymers. Several studies revealed that fluorinated polymers have higher PCEs compared with non-fluorinated analogues. It has been reported that conjugated polymers based on 3,3′-difluoro-2,2′-bithiophene and alkylthio-substituted theno[3,3-*b*]thiophene, exhibited a PCE of 5.2% [20]. Very recently, Ohkita et al. reported naphtho[1,2-*c*:5,6-*c*]bis[1,2,5]thiadiazole (NTz)-based copolymers with fluorinated bithiophene, namely PNTz4TF2 gave a PCE of 10.5% [21]. Importantly, the number of substituted fluorine atoms in conjugated polymers can affect the photovoltaic performances, although, in few cases, it has been shown to be non-beneficial due to the larger aggregation of polymer, poor solubility, and fluorophobicity effect. Won Ho et al. synthesized conjugated polymer comprised of 3,3′-difluoro-2,2′-bithiophene and 2,1,3-benzothiadiazole (BT) with different numbers of fluorine substitution and demonstrated that mono-fluorinated BT showed a superior PCE of 9.14% compared with the di-fluorinated BT with a PCE of 6.43% due to lower bimolecular charge recombination and high hole mobility [22]. Though this report is impressive on the degree of fluorination, there is still a need for studies to explain the effect of fluorination on donor/acceptor (D/A) units of polymer with different chemical structures.

Recently, selenium-based electron-rich heteroaromatic building blocks such as selenophene, benzodiselenophene, and selenolo[3,2-*b*]thiophene were employed to build conjugated polymers for polymer solar cells [23] and organic field-effect transistors [24]. The incorporation of Se atom in polymer backbone reduces the bandgap and absorbs red light region of the solar spectrum. Moreover, the strong Se–Se interactions due to its polarizable nature frequently leads to favorable nano-scale morphologies with improved charge mobility, high short-circuit current density and high hole mobility. Several studies have been reported on the advantages of using Se-containing narrow bandgap polymers [23,25]. For example, the use of benzoselenadiazole (BSe), an analogue of electron acceptor benzothiadiazole (BT), as electron-deficient building block effectively lowers the HOMO energy level, broadens light absorption spectrum and increases hole mobility. Keisuke et al. reported copolymer PBDT–DTBSe based on benzodithiophene (BDT) as donor moiety and benzoselenadiazole (BSe) as acceptor unit that exhibits broad absorption spectrum and the solar cells using such polymer as electron donor had a PCE of 5.18%, which was slightly higher PCE than that of the benzothiadiazole analogue [26]. Hwang et al. synthesized PEHBDT–DFDTBSe copolymer with a narrow bandgap of 1.69 eV and a PCE of 5.74% [27]. Chen et al. reported PBDT–T–FBSe and PIDT–T–FBSe both of which showed broad absorption spectrum with a PCE of 5.0% and 4.65%, respectively [28]. In spite of the above-mentioned advantages, there are still drawbacks, especially the poor performance of the devices. Hence, studying the effect of substitution units and their implication in optimizing device structure is still in need.

In this contribution, we present a newly designed two-dimensional conjugated copolymer containing 4,7-di(thiophen-2-yl)benzo[*c*][1,2,5]selenodiazole (DTBSe) acceptor unit, 3,3′-difluoro-2,2′-bithiophene (2TF), and electron-donating conjugated vinyl-terthiophene (VTT) for efficient polymer solar cells. The incorporation of vinyl-terthiophene (VTT) along with two 2-octyldodecyl solubilizing groups onto the benzoselenodiazole-thiophene backbone as side chains can increase the solubility, red-shift the absorption spectrum, lower the HOMO and LUMO energy levels and retain excellent crystallinity. Optical and electrochemical properties of PDTBSeVTT-2TF copolymer were systematically investigated. PDTBSeVTT-2TF exhibits a small bandgap with broad absorption in the NIR region and deep HOMO and LUMO energy levels. Furthermore, the best PSC device based on PDTBSeVTT-2TF:PC_71_BM (1:1.2, *w/w*) with 3% diiodooctane (DIO) processed at 65 °C showed a promising PCE of 5.11% with a *J_sc_* of 10.99 mA/cm^−2^ under AM1.5G illumination at one sun intensity.

## 2. Experimental Section

### 2.1. Materials

All solvents and reagents were commercially purchased from Aldrich (UNI-ONWARDCO., New Taipei City, Taiwan), Alfa Aesar (Haverhill, MA, USA) or Across (Dinhaw Enterprise Co., New Taipei City, Taiwan) and used as purchased. PC_70_BM (> 99%) was purchased from Nano-C (Westwood, MA, USA) and used as received. 5,5′-Bis-(trimethylstannyl)-3,3′-difluoro-2,2′-bithiophene monomer M2 was purchased and used; 4,7-dibromo-5-methylbenzo[*c*][1,2,5]thiadiazole **1** [29], 5-tributylstannyl-2-thiophene **6** [30], tributy(4-(2-octyldodecyl)thiophene-2-yl)stannane **9** [31], 2,5-dibromothiophene-3-carbaldehyde **10** [32] were prepared according to the literature procedures.

### 2.2. Instruments and Characterizations 

Still coupling reactions were conducted in Anton Paar Monowave 300 microwave reactor (Graz, Austria). ^1^H and ^13^C NMR spectra were recorded using the chloroform-d (CDCl_3_) and Dimethylsulfoxide-d_6_ (DMSO-d_6_) at 400 MHz on Bruker DRX-400 spectrometer (Bruker Taiwan Co., Taipei, Taiwan). UV–Vis absorption spectra in solution and thin film were taken using a JASCO MD-2010 spectrometer (JASCO, Tokyo, Japan). A potentiostat from CH instruments (model: CHI6147, Austin, TX, USA) was employed to record cyclic voltammograms of the studied compounds in a sample vial equipped with a platinum plate, a platinum wire and a Ag/AgCl as the working, counter and reference electrodes, respectively. The measurements were carried out at a scan rate of 100 mV s^−1^ using tetrabutylammonium hexafluorophosphate (TBAPF_6_) as supporting electrolyte and acetonitrile as solvent. The reference electrode was calibrated with ferrocene internal standard. TEM images were captured using a Hitachi H-7100 transmission electron microscope, (Hitachi Ltd, Tokyo, Japan).

### 2.3. Fabrication and Measurement of Polymer Solar Cells

Polymer solar cells were fabricated in an inverted device configuration of ITO/ZnO/PDTBSeVTT-2TF:PC_71_BM/V_2_O_5_/Ag. The ITO glass substrates were cleaned by a sequential ultrasonic treatment in detergent, deionized water, acetone, and isopropanol for 20 min each and then dried using nitrogen gun. For the inverted PSCs, Zinc acetate dihydrate (Zn(OAc)_2_·2H_2_O) was dissolved in 2-methoxyethanol (100 mg/mL) and ethanolamine was spin-coated on pre-cleaned ITO substrates and heated at 160 °C for 10 min in open air to form the Zinc oxide (ZnO) layer with a thickness of 20 nm. The PDTBSeVTT-2TF:PC_71_BM (1:1.2, *w/w*) were dissolved in a solvent mixture of chlorobenzene/1,8-diiodooctane (DIO) (97/3, *v/v*) at 50 °C and 65 °C for 12 h respectively, and filtered through a 0.45 μm filter and spin-coated at 1500 rpm for 60 s on the ITO/ZnO layer. The anode made of V_2_O_5_ (7 nm) and Ag (90 nm) was evaporated through a shadow mask under high vacuum pressure of ca. 10^−6^ Pa. Each sample consisted of six independent pixels with an active area of 0.07 cm^2^. The current density–voltage (*J*–*V*) characteristics of solar cells were recorded on a computer-controlled Keithley 2400 source measurement system (Keithly, Cleveland, OH, USA) under AM1.5G illumination at 100 mW cm^−2^, which was adjusted using a pre-calibrated mono-Si reference cell with a KG5 filter purchased from PV Measurements Inc., Boulder, CO, USA.

### 2.4. Synthesis of Monomers and Polymers

#### 2.4.1. Synthesis of 3,6-Dibromo-4-methylbenzene-1,2-diamine (**2**)

To a stirred solution of 4,7-dibromo-5-methylbenzo[*c*][1,2,5]thiadiazole **1** (2 g, 6.55 mmol) in EtOH (250 mL) at 0 °C under N_2_ was added sodium borohydride (4.96 g, 131.14 mmol) portion-wise. After stirring at 0 °C for 10 min, reaction mixture was stirred for 12 h at room temperature. After removal of the solvent under reduced pressure, water (200 mL) was added, and the mixture was extracted with ether twice (100 mL). The organic layer was washed with brine and dried over anhydrous MgSO_4_. Combined ether layer was evaporated to give pale yellow solid **2** (1.43 g, yield 78%). ^1^H NMR (400 MHz, CDCl_3_, δ, ppm): 6.85 (s, 1H), 3.61 (brs, 4H), 2.25 (s, 3H). ^13^C NMR (100 MHz, CDCl_3_, δ, ppm): 134.19, 130.69, 129.77, 123.05, 112.18, 109.65, 22.53.

#### 2.4.2. Synthesis of 4,7-Dibromo-5-methyl-2,1,3-benzoselenadiazole (**3**)

To a stirred solution of 3,6-dibromo-4-methylbenzene-1,2-diamine **2** (1.3 g, 4.64 mmol) in refluxing ethanol (50 mL) was added a solution of selenium dioxide (0.61 g, 5.10 mmol) in hot water (20 mL). The mixture was heated to reflux for 12 h. Yellow solid formed was filtered and washed with ethanol (5 × 50 mL) to afford 4,7-dibromo-5-methyl-2,1,3-benzoselenadiazole **3** as a yellow powder (1.34 g yield 82%). ^1^H NMR (400 MHz, CDCl_3_, δ, ppm): 7.66 (s, 1H), 2.52 (s, 3H). ^13^C NMR (100 MHz, CDCl_3_, δ, ppm): 157.53, 155.92, 140.12, 135.11, 116.48, 114.95, 22.54.

#### 2.4.3. Synthesis of 4,7-Dibromo-5-(bromomethyl)benzo[*c*][1,2,5]selenadiazole (**4**)

A mixture of 4,7-dibromo-5-methyl-2,1,3-benzoselenadiazole **3** (1.1 g, 3.1 mmol), *N*-bromosuccinimide (0.60 g, 3.41 mmol) and catalytic amount of AIBN (30 mg) were dissolved in chloroform (50 mL) in a two-necked round-bottomed flask. The mixture was heated to reflux for 24 h under N_2_. After cooling to room temperature, water (100 mL) was added, and the mixture was extracted with chloroform (2 × 50 mL). Combined organic layer was washed with brine and dried over anhydrous MgSO_4_. After evaporation, the residue was purified by silica gel column chromatography using (EtOAc/hexane, 1:4) as an eluent to give 4,7-dibromo-5-(bromomethyl)benzo[*c*][1,2,5]selenadiazole **4** as a pale-yellow solid (0.82 g, yield 61%). ^1^H NMR (400 MHz, DMSO-d_6_, δ, ppm): 8.07 (s, 1H), 4.83 (s, 2H). ^13^C NMR (100 MHz, DMSO-d_6_, δ, ppm): 156.70, 155.72, 138.87, 133.32, 118.30, 115.81, 33.39.

#### 2.4.4. Synthesis of Diisopropyl(4,7-dibromobenzo[*c*][1,2,5]selenadiazol-6-yl) Methylphosphonate (**5**)

4,7-Dibromo-5-(bromomethyl)benzo[*c*][1,2,5]selenadiazole (0.8 g, 1.84 mmol) and triisopropyl phosphite (3.84 g, 18.47 mmol) were mixed in a 25 mL two-neck round-bottomed flask equipped with a condenser and heated to 160 °C for 6 h. The crude product was directly purified by column chromatography using (EtOAc/hexane, 3:2), as an eluent to give the title compound **5** as a yellow solid (0.82 g, yield 86%). ^1^H NMR (400 MHz, CDCl_3_, δ, ppm): 7.89 (s, 1H), 4.71–4.60 (m, 2H), 3.45 (d, 2H, *J* = 22.8 Hz), 1.29 (d, 6H, *J* = 6.0 Hz), 1.25 (d, 6H, *J* = 6.4 Hz). ^13^C NMR (100 MHz, CDCl_3_, δ, ppm): 157.39, 155.99, 135.26 (d, *J_cp_* = 10.24 Hz), 134.58, 117.75 (d, *J_cp_* = 12.12 Hz), 115.10, 71.34 (d, *J_cp_* = 6.84 Hz), 35.62, 34.23, 23.98, 23.94, 23.90, 23.85.

#### 2.4.5. Synthesis of Diisopropyl (4,7-di(Thiophen-2-yl)benzo[*c*][1,2,5]selenadiazol-6-yl) Methylphosphonate (**7**)

A 30 mL glass vial with a stir bar was charged with diisopropyl (4,7-dibromobenzo[*c*][1,2,5]selenadiazol-6-yl) methyl phosphonate **5** (0.8 g, 1.56 mmol) 2-tributylstannyl-thiophene (1.44 g, 3.59 mmol), catalytic amount of Pd(PPh_3_)_4_ (10 mg) and anhydrous toluene (15 mL). The glass vial was then placed into a microwave reactor and irradiated to 210 °C for 1 h. After evaporation of the solvent under reduced pressure, the crude mixture was purified by column chromatography using (EtOAc/hexane, 2:3) as an eluent to afford compound **7** as a yellow solid (0.63, yield 77%). ^1^H NMR (400 MHz, CDCl_3_, δ, ppm): 8.05–8.03 (m, 2H), 7.52 (d, 1H, *J* = 5.12 Hz), 7.44–7.41 (m, 2H), 7.18–7.15 (m, 2H), 4.72–4.64 (m, 2H), 3.30 (d, 2H, *J* = 22.84 Hz), 1.29 (d, 6H, *J* = 6.2 Hz), 1.20 (d, 6H, *J* = 6.16 Hz). ^13^C NMR (100 MHz, CDCl_3_, δ, ppm): 161.17, 156.67, 139.11, 136.37, 133.41 (d, *J_cp_* = 8.55 Hz), 129.62, 128.78, 127.89, 127.66, 127.48, 127.33, 127.19, 127.10, 126.87, 70.90 (d, *J_cp_* = 6.91 Hz), 33.31, 31.93, 24.01, 23.96, 23.92, 23.89.

#### 2.4.6. Synthesis of Diisopropyl (4,7-bis(5-Bromothiophen-2-yl)benzo[*c*][1,2,5]selenadiazol-6-yl) Methylphosphonate (**8**)

A mixture of diisopropyl(4,7-di(thiophen-2-yl)benzo[*c*][1,2,5]selenadiazol-6-yl)methylphosphonate **7** (0.6 g, 1.14 mmol) and NBS (0.4 g, 2.28 mmol) in DMF (30 mL) was stirred at room temperature for 12 h. Reaction mixture was poured into ice water (100 mL) and extracted chloroform (2 × 50 mL). Combined organic layer was dried over MgSO_4_. After removal of the solvent under reduced pressure, the residue was purified by column chromatography using (EtOAc/hexane, 1:1) as an eluent to afford compound **8** as yellow solid (0.55 g, yield 70%). ^1^H NMR (400 MHz, CDCl_3_, δ, ppm): 7.95 (d, 1H, *J* = 1.72 Hz), 7.71 (d, 1H, *J* = 4.0 Hz), 7.24 (d, 1H, *J* = 3.76 Hz), 7.11 (d, 1H, *J* = 3.8 Hz), 7.09 (d, 1H, *J* = 3.96 Hz) 4.72–4.64 (m, 2H), 3.25 (d, 2H, *J* = 22.84 Hz), 1.30 (d, 6H, *J* = 6.2 Hz), 1.20 (d, 6H, *J* = 6.2 Hz). ^13^C NMR (100 MHz, CDCl_3_, δ, ppm): 160.51, 156.14, 140.16, 137.86, 133.48 (d, *J_cp_* = 9.71 Hz), 130.30, 130.11, 129.72, 128.03, 127.41, 126.81, 126.38 (d, *J_cp_* = 10.45 Hz), 115.54, 114.49, 71.10 (d, *J_cp_* = 6.83 Hz), 33.35, 31.96, 24.01, 23.98, 23.94, 23.89.

#### 2.4.7. Synthesis of 2,5-bis(4-(2-Octyldodecyl)thiophen-2-yl)thiophene-3-carbaldehyde (**11**)

A 30 mL glass vial equipped with a stir bar was charged with 2,5-dibromothiophene-3-carbaldehyde **10** (0.8 g, 2.9 mmol) and 5-tributylstannyl-3-(2-Octyldodecyl)thiophene **9** (5.08 g, 7.76 mmol), catalytic amount of Pd(PPh_3_)_4_ (10 mg) and anhydrous toluene (15 mL). The glass vial was then placed into a microwave reactor and irradiated to 210 °C for 1 h. After removal of the solvent under reduced pressure, the mixture was purified by column chromatography using hexane as an eluent to afford compound **11** as a yellow liquid (1.1 g yield 44%). 1H NMR: ^1^H NMR (400 MHz, CDCl_3_, δ, ppm):10.06 (s, 1H), 7.48 (s, 1H), 7.06 (s, 1H), 7.00 (s, 1H), 6.98 (s, 1H), 6.80 (s, 1H), 2.53 (d, 2H, *J* = 6.76 Hz), 2.48 (d, 2H, *J* = 6.76 Hz), 1.59 (br s 2H), 1.24–1.21 (m, 64H), 0.86–085 (m, 12). ^13^C NMR (100 MHz, CDCl_3_, δ, ppm): 185.05, 146.21, 143.38, 143.00, 137.31, 136.81, 134.9, 131.60, 130.82, 126.54, 124.16, 121.83, 121.16, 38.81, 38.74, 34.84, 33.23, 31.83, 29.89, 29.27, 26.52, 22.60, 14.02.

#### 2.4.8. 5-((*E*)-2-(2,5-bis(4-(2-Octyldodecyl)thiophen-2-yl)thiophen-3-yl)vinyl)-4,7-bis(5-bromo thiophen-2-yl)benzo[*c*][1,2,5]selenadiazole **M1**

Diisopropyl (4,7-bis(5-bromothiophen-2-yl)benzo[*c*][1,2,5]selenadiazol-6-yl)methyl phosphonate **8** (0.7 g, 1.02 mmol) was taken in a 100 mL Schlenk flask and degassed via vacuum-N_2_ cycle three times and anhydrous THF (50 mL) was added and cooled to 0 °C. To this was added sodium *tert*-butoxide (0.10 g, 1.02 mmol). After stirring for 1 h, 2,5-bis(4-(2-octyldodecyl)thiophen-2-yl)thiophene-3-carbaldehyde **11** (0.96 g, 1.02 mmol) which was dissolved in anhydrous THF (10 mL) was added. The reaction mixture was allowed to stir at room temperature and stirred overnight. Reaction mixture was poured into ammonium chloride aqueous solution and extracted ethyl acetate (2 × 50 mL). Combined organic layer dried over MgSO_4_. After removal of the solvent, the residue was purified by column chromatography (CHCl_3_/hexane, 2:8) to afford monomer **M1** as a red viscous liquid (1.06 g, yield 77%). NMR (400 MHz, CDCl_3_, δ, ppm ): 7.91 (s, 1H), 7.62 (d, 1H, *J* = 3.88 Hz), 7.44 (d, 1H, *J* = 16.08 Hz), 7.19 (d, 1H, *J* = 16.12 Hz), 7.14–7.12 (m, 2H), 7.05–7.03 (m, 2H), 6.97–6.96 (m, 3H), 6.79 (s, 1H), 2.57 (d, 2H, *J* = 6.52 Hz), 2.50 (d, 2H, *J* = 6.6 Hz), 1.62 (brs, 2H), 1.25–1.21 (m, 64H), 0.85–0.82 (m, 12H). ^13^C NMR (100 MHz, CDCl3): 160.04, 156.56, 142.89, 140.43, 138.15, 136.5, 136.39, 135.71, 135.35, 134.28, 133.71, 130.82, 130.23, 129.53, 128.85, 127.21, 126.83, 126.05, 124.36, 123.67, 122.34, 121.42, 120.55, 115.33, 38.83, 38.77, 34.99, 34.90, 33.32, 33.26, 31.86, 29.98, 29.63, 29.60, 29.30, 26.56, 22.63, 14.06.

#### 2.4.9. Synthesis of Polymer PDTBSeVTT-2TF

To a mixture of monomer (460.4 mg, 0.34 mmol) **M1**, 5,5′-Bis-(trimethylstannyl)-3,3′-difluoro-2,2′-bithiophene monomer (181.6 mg, 0.34 mmol) **M2** and Pd_2_dba_3_ (9.5 mg, 0.01 mmol)/P(*o*-tol)_3_ (6.3 mg, 0.02 mmol) in a dry 10 mL microwave vial (G10) was added dry toluene (4.3 mL). The vial was sealed under nitrogen and then irradiated by microwave. The heating condition was raise temperature from room temperature to 210 °C as fast as possible; hold time 120 min; cool down to 55 °C. The crude polymer was dissolved by toluene, then precipitated with methanol and collected through a Soxhlet thimble filtration, which was further subjected to successive Soxhlet extraction with methanol, acetone, hexanes, dichloromethane and chloroform. The final polymer was collected from chloroform-soluble fraction by rotary evaporation and dried in vacuum for 12 h at 60 °C, yielding PDTBSeVTT-2TF (380 mg, yield ~80%).

## 3. Results and Discussion

### 3.1. Synthesis and Physical Properties of Polymer PDTBSeVTT-2TF

**Scheme 1:**
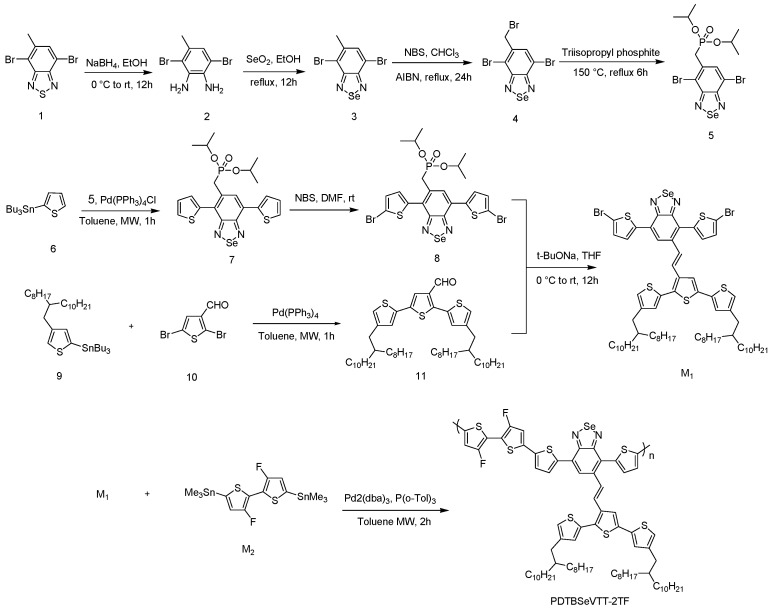
Synthetic routes for monomer **M1** and copolymer PDTBSeVTT-2TF.

The synthetic routes for preparing monomer **M1**, copolymer PDTBSeVTT-2TF and key intermediates **8** and **11** are summarized in Scheme 1. 4,7-Dibromo-5-methylbenzo[*c*][1,2,5]thiadiazole **1** [29] was prepared according to a procedure reported elsewhere. Reduction of compound **1** with NaBH_4_ (20 eq) led to the diamine compound **2**, which was then oxidized with SeO_2_ to form 4,7-dibromo-5-methyl-2,1,3-selenodiazole **3**. Bromination of compound **3** with *N*-bromosuccimide (NBS) and catalytic AIBN afforded 4,7-dibromo-5-bromomethyl-2,1,3-selenodiazole **4**, which was treated with triisopropyl phosphite to give phosphonic acid diethyl ester **5**. Compound **5** was reacted with 5-tributylstannyl-2-thiophene **6** [30] and catalytic amount of Pd(PPh_3_)_4_ under Stille coupling microwave condition to afford phosphonic acid diisopropyl ester **7**. Compound **7** was brominated by using *N*-bromosuccimide to afford Compound **8**. Terthiphene aldehyde intermediate **11** was synthesized by reacting 2,5-dibromothiophene-3-carbaldehyde **10** [32] with 2.5 equivalents of tributy(4-(2-octyldodecyl)thiophene-2-yl)stannane **9** [31] using Pd(PPh_3_)_4_ as catalyst under Stille coupling microwave conditions to produce corresponding aldehyde intermediate **11**. The new monomer **M1** was synthesized via the Wittig–Horner reaction through the coupling of phosphonic acid diisopropyl ester **8** and aldehyde **11.** The chemical structure of monomer **M1** was confirmed by ^1^H and ^13^C NMR spectra.

The microwave-assisted (MW) reaction has become a more and more important route to synthesize polymers with better yield, higher molecular weight and accelerated reaction rate [33]. The copolymer PDTBSeVTT-2TF was synthesized from 5,5′-bis-(trimethylstannyl)-3,3′-difluoro-2,2′-bithiophene monomer **M2** and dibromo monomer **M1** by the microwave Stille coupling reaction using Pd_2_(dba)_3_/P(*o*-tolyl)_3_ as catalyst. After the reaction, we observed trace amounts of decomposed palladium catalyst on the wall of the reactor vial as reported by Scherf et al. [33]. The crude product was precipitated with methanol and filtered through a Soxhlet thimble which was further subjected to successive Soxhlet extractions with methanol, acetone, hexane and dichloromethane to remove small molecules, oligomers and decomposed metal impurities. Finally, the chloroform-soluble product was dried by a rotary evaporator to afford PDTBSeVTT-2TF with a yield of ~80%. The synthesized polymer is soluble in various organic solvents such as chloroform, chlorobenzene, toluene, tetrahydrofuran (THF), and *o*-dichlorobenzene (*o-*DCB) and can form uniform thin film upon spin-coating. The number-average molecular weight (*M*_n_) and polydispersity index (PDI) of the resulting PDTBSeVTT-2TF were found to be 74.5 kDa and 3.34, respectively, as measured by gel permeation chromatography (GPC) at 40 °C using THF as eluent and polystyrene samples as standard, as shown in Table 1.

**Figure 1:**
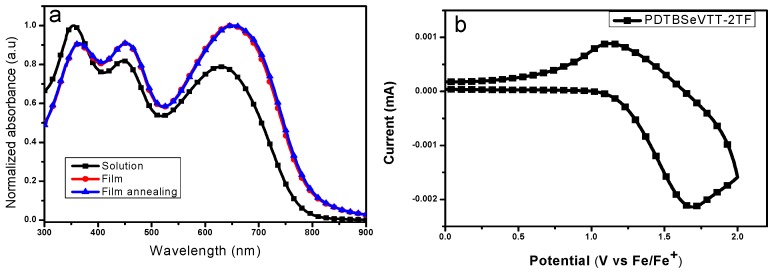
(**a**) Ultraviolet–Vis absorption spectra of PDTBSeVTT-2TF in dilute chlorobenzene solution at room temperature and as thin film before and after thermal annealing at 120 °C for 15 min; (**b**) CV plot of PDTBSeVTT-2TF measured in 0.1 M TBAPF_6_/ACN at a scan rate of 100 mV·s^−1^.

Ultraviolet–Visible analysis was carried out to determine the electron-withdrawing effect of fluorine atoms and vinyl-terthiophene side chains on the optoelectronic properties of PDTBSeVTT-2TF. Figure 1 displays the UV absorption spectra of the polymer as a diluted chlorobenzene solution (1 × 10^−4^ M) and as a spin-coated thin film on a glass substrate. The corresponding maximum absorption wavelengths (λ_max_) and optical bandgap are summarized in Table 2. As shown in Figure 1, PDTBSeVTT-2TF exhibits similar absorption spectral features with three major absorption peaks across wavelength region 300–800 nm in solution and solid film. However, the absorption bands of polymer film were broader and red-shifted compared with those of the corresponding solution, indicating that such polymer film possesses intramolecular charge transfer between the vinyl-terthiophene and benzoselenodiazole units as well as strong intermolecular π–π interaction between backbones, resulting in more ordered structural organization of polymer chains. In addition, the absorption spectra of thermally annealed film of PDTBSeVTT-2TF at 120 °C for 15 min is almost identical to that of the as-cast films, demonstrating that the polymer backbone has attained desirable π–π stacking before thermal treatment [34]. The optical bandgap (*E*_g_^op^) of PDTBSeVTT-2TF is determined to be 1.57 from the absorption edges (λ_edge_) of the solid film. Such wide absorption band and narrow bandgap are beneficial to achieving high short-circuit current (*J_sc_*).

The cyclic voltammetry (CV) experiment was performed to attain molecular energy levels of PDTBSeVTT-2TF. CV measurements of the as-cast film of PDTBSeVTT-2TF on a Pt electrode was carried out at a scan rate of 100 mV·s^−1^ using 0.1 M TBAPF_6_/acetonitrile solution as the electrolyte and a Pt wire and Ag/AgCl electrode as the counter and reference electrodes, respectively. Figure 1b plots the CV curve and the detailed data are presented in Table 2. The onset oxidation potential of PDTBSeVTT-2TF is at 1.19 eV and the HOMO energy of the polymer was calculated from the onset oxidation potential (*E*_onset,ox_) according to the equation: HOMO = −e(*E*_onset,ox_ − *E*_(Fc/Fc+)_ + 4.80) (eV) [35], where the potential (*E*_(Fc/Fc+)_) of the external standard of ferrocene/ferrocenium redox couple under the same experimental conditions was −0.42 eV. The determined HOMO value is −5.57 eV. Then, the LUMO level was calculated from the optical bandgap and the HOMO level, according to the equation: LUMO = HOMO + *E*_g_^opt^, resulting in a LUMO level of −4.0 eV, which lies slightly above the LUMO of PC_70_BM (−4.2 eV) [36].

### 3.2. Photovoltaic Properties

**Figure 2:**
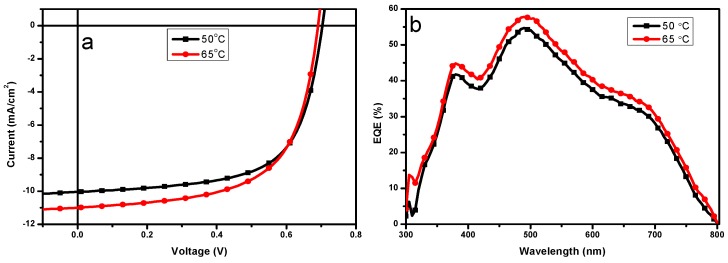
(**a**) The current density–voltage (*J*–*V*) curves; (**b**) EQE spectra of BHJ solar cells fabricated from the PDTBSeVTT-2TF:PC_71_BM blend solution at 50 and 65 °C. EQE = external quantum efficiencies; BHJ = bulk heterojunction.

The photovoltaic properties of the bulk heterojunction solar cells based on PDTBSeVTT-2TF was investigated by using [6,6]-phenyl-C71-butyric acid methyl ester as electron acceptor and the cells were fabricated in an inverted device configuration, ITO/ZnO/PDTBSeVTT-2TF:PC_71_BM/V2O5/Ag. First of all, we varied the ratio of donor/acceptor (D/A) and the amount of additive in the polymer/fullerene blend solution where chlorobenzene is the solvent (Figures S1 and S2, Tables S1 and S2 in Supporting information), and learned that the optimal condition is 1:1.2 weight ratio of D/A with 3 wt % of 1,8-diiodooctane (DIO) as processing additive. Moreover, the blend solution of PDTBSeVTT-2TF:PC71BM was stirred and kept at two different temperatures—50 and 65 °C before spin coating. Figure 2 plots the current density–voltage (*J*–*V*) curves of these devices under AM1.5G illumination with an intensity of 100 mW cm^−2^. The corresponding average photovoltaic parameters, including open-circuit voltage (*V_oc_*), short-circuit current (*J_sc_*), fill factor (FF), power conversion efficiency (PCE), series resistance (*R_s_*) and shunt resistance (*R_sh_*), are presented in Table 3.

The solar cell based on PDTBSeVTT-2TF/PC_71_BM blend solution processed at 65 °C showed an average PCE of 5.11% with a *V_oc_* of 707 mV, a *J_sc_* of 10.99 mA·cm^−2^ and a FF of 62.01%, while the device processed at 50 °C exhibited a relatively lower PCE of 4.69% with a *V_oc_* of 711 mV, a *J_sc_* of 10.10 mA/cm^−2^ and a FF of 62.73%. As shown in Figure 2a, the *J_sc_* of the 65 °C-device is about 8% higher than that of the 50 °C-cell. This observation reveals that the PDTBSeVTT-2TF may form clusters in chlorobenzene at room temperature and dissolute into smaller aggregates and individual chains at higher temperature, which improves the miscibility between PDTBSeVTT-2TF donor and PC_70_BM acceptor and increases the interface of the two materials, leading to the enhancement of photocurrent. However, both devices have comparable *V_oc_*, which is primarily determined by the energy difference between the HOMO of the polymer and the LUMO of the fullerene acceptor.

To verify the *J_sc_* values obtained from *J–V* measurements, the external quantum efficiencies (EQEs) of the solar cells fabricated from the PDTBSeVTT-2TF/PC71BM blend solution at two different temperatures were measured, and are shown in Figure 2b. Basically, both EQE spectra have very similar curve shapes between 300–800 nm and comprise one major and two minor bands; the 50 °C-device exhibits lower EQE values over the entire wavelength region from 350 to 850 nm than those of the 65 °C-device. The integral current densities obtained from the EQE curves of the former and the latter cells are 9.80 and 10.56 mA/cm^2^, respectively, which are in good agreement with those determined from the *J*–*V* measurements. These findings suggest that the enhanced *J_sc_* and EQE values for the 65 °C-device are due to the formation of a better morphology of the active layer.

**Figure 3:**
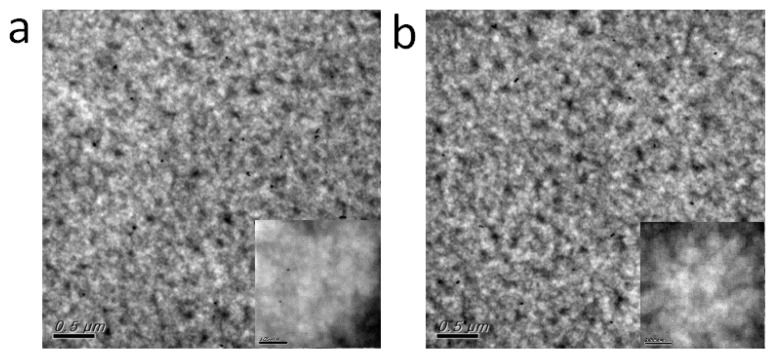
TEM images of PDTBSeVTT-2TF:PC_71_BM blend films which were prepared from the blend solution in ODCB at (**a**) 50 °C and (**b**) 65 °C.

Transmission electron microscopy (TEM) was used to investigate the effect of solution temperature on the nano-morphology of the PDTBSeVTT-2TF/PC_71_BM blend films. Figure 3a,b shows the TEM images of the solid films prepared from the blend solution at 50 and 65 °C, respectively. The bright areas represent the PDTBSeVTT-2TF-rich domains and the dark regions are the PC_71_BM-rich phases. Both pictures exhibit comparable morphology with a nano-scale bulk heterojunction structure. However, the high magnification images in the inserts indicate the formation of relatively large PDTBSeVTT-2TF-rich domains in Figure 3a; this result demonstrates that the higher solution temperature may prevent the massive aggregation of PDTBSeVTT-2TF into big clusters, facilitating the diffusion of PC_71_BM molecules into the polymer matrix and thus reducing the domain sizes.

**Figure 4:**
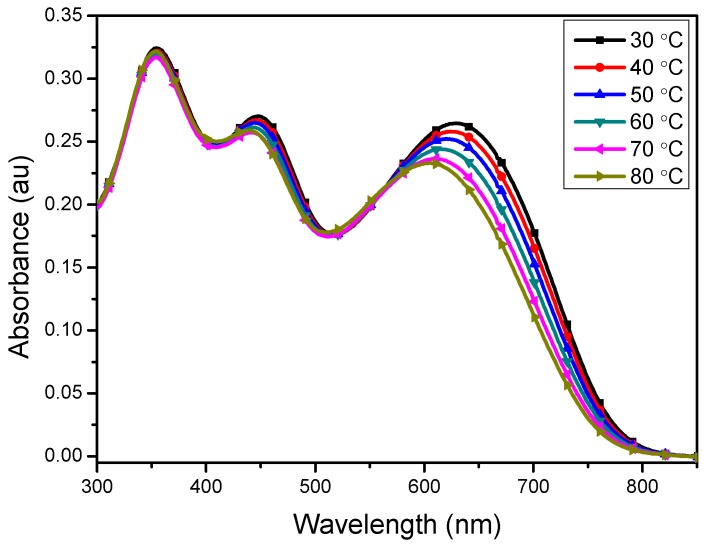
Temperature-dependent UV–Vis absorption spectra of PDTBSeVTT-2TF in diluted chlorobenzene solution at 1 × 10^−4^ M.

A series of temperature-dependent UV–Vis absorption measurements were performed to study the aggregation behavior of PDTBSeVTT-2TF in diluted chlorobenzene solution at 1 × 10^−4^ M from 30 to 80 °C. As shown in Figure 4, the absorption spectra of PDTBSeVTT-2TF exhibit gradually decreased intensity in their π–π stacking absorption peak and blue-shifted absorption wavelengths with increasing temperature from 30 to 80 °C, indicating that the polymer chains consistently disaggregated with the destruction of coplanarity and the increase of solubility due to the enhanced molecular thermal motion. These findings are totally consistent with the previously discussed TEM results and support the previous assumption about the aggregation of PDTBSeVTT-2TF in ODCB at lower solution temperature.

**Figure 5:**
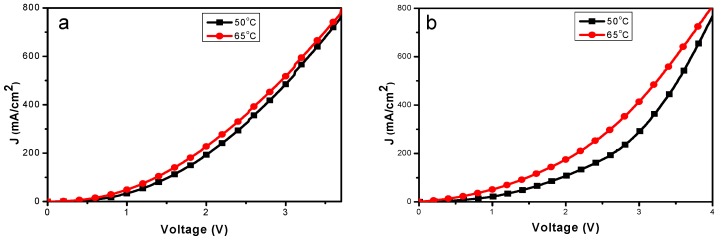
(**a**,**b**) Measured space-charge-limited *J*–*V* curves of the (**a**) hole-only and (**b**) electron-only devices based on PDTBSeVTT-2TF:PC_71_BM blend films processed at different temperatures.

To further study how the temperature of the blend solution affects photovoltaic performances of PDTBSeVTT-2TF/PC_71_BM solar devices in terms of the FF, we measured both hole and electron mobilities of the blend films using the space-charge-limited current (SCLC) method with device structures of ITO/ZnO/PDTBSeVTT-2TF:PC_71_BM/Ca/Al for electron-only and ITO/PEDOT:PSS/PDTBSeVTT-2TF:PC_71_BM/Au for hole-only devices, respectively. Figure 5 plots the space-charge-limited *J*–*V* curves of these devices and Table 4 lists the determined values of the hole and electron mobilities. Both electron (µ_e_ = 2.65 × 10^−4^ cm^−2^·V^−1^·s^−1^) and hole (µ_h_ = 3.11 × 10^−4^ cm^−2^·V^−1^·s^−1^) mobilities of the PDTBSeVTT-2TF:PC_71_BM blend film processed from a 65 °C-solution are slightly higher than those (µ_e_ = 2.0156 × 10^−4^; µ_h_ = 1.915 × 10^−4^ cm^−2^·V^−1^·s^−1^) of the film processed from a 50 °C-solution, but they are in the same order of magnitude. Therefore, both solar devices exhibit comparable series resistance (*R_s_*) and shunt resistance (*R_sh_*). Furthermore, the ratio of hole to electron mobilities (μ_h_/μ_e_ = 0.95) in the 50 °C-film is more balanced than that (μ_h_/μ_e_ = 1.17) of the 65 °C-film, leading to an improved FF for the 50 °C-device.

## 4. Conclusions

In this work, we designed and synthesized new two-dimensional conjugated polymer using fluorinated bithiophene (2TF) as donor and 4,7-di(thiophen-2-yl)benzo[*c*][1,2,5]selenodiazole (DTBSe) as acceptor units, anchored with electron-donating vinyl-terthiophene (VTT) conjugated side chains, each of which comprises two 2-octyldodecyl groups to improve their solubility. The results from UV-absorption and CV measurements clearly demonstrate that this selenium-containing fluorinated polymer, PDTBSeVTT-2TF, exhibited broad absorption range, strong π–π stacking effect in solid film and a narrow optical bandgap of 1.57 eV with a deep HOMO of −5.57 eV. Bulk heterojunction polymer solar cells (PSCs) were fabricated with an inverted device structure of ITO/ZnO/PDTBSeVTT-2TF:PC_71_BM/V_2_O_5_/Ag. The active layers were produced from the blend solution at 50 and 65 °C. The device processed from the 65 °C solution exhibited an improved power conversion efficiency. This enhancement primarily because the increased thermal motion of polymer chains dissociates the PDTBSeVTT-2TF clusters into small aggregates and single macromolecules that facilitate the insertion of PC_71_BM into polymer matrix to form nano-scale domains, thus increasing the D/A interface and enhancing the photocurrent.

## Figures and Tables

**Table 1:** Molecular weight characteristics of PDTBSeVTT-2TF.

Polymer	Yield (%)	*M*_w_ (kDa)	*M*_n_ (kDa)	PDI
PDTBSeVTT-2TF	80	249.6	74.5	3.34

**Table 2:** Optical and electrochemical properties of PDTBSeVTT-2TF.

Polymer	λ_max_ (nm)			λ_onset_ (nm)	*E*_g_^opt^ (eV) Film ^e^	*E*_onset,ox_ (V)	HOMO (eV) ^f^	LUMO (eV) ^g^
	Solution ^a^	Film ^b^	Film Annealed ^c^	Film Annealed ^d^				
**PDTBSeVTT-2TF**	356, 447, 629	365, 451, 647	367, 453, 651	796	1.57	1.19	−5.57	−4.0

^a^ Measured in dilute chlorobenzene solution at room temperature; ^b^ Evaluated on glass substrates by polymer spin coated from a chlorobenzene solution (10 mg/L); ^c^ Thermal annealing at 120 °C for 15 min; ^d^ Absorption edge of film; ^e^ Calculated from the onset wavelength of the absorption spectra: *E*_g_^opt^ = 1240/λ_onset_; ^f^ HOMO energy levels were calculated by CV using HOMO = −e(*E*_onset,ox_ − *E*_(Fc/Fc+)_ + 4.80) (eV); ^g^ LUMO = HOMO + *E*_g_^opt^ (eV).

**Table 3:** Photovoltaic properties of PDTBSeVTT-2TF:PC_71_BM solar cells with different processing temperature under the illumination of AM1.5G at 100 mW·cm^−2^.

Processing Temperature	*V_oc_* (mV)	*J_sc_* (mA/cm^2^)	FF (%)	PCE (%)	*R*_s_ (Ωcm^2^)	*R*_sh_ (Ωcm^2^)
50 °C	711.25 ± 4.39	10.10 ± 0.22	62.73 ± 1.12	4.50 ± 0.13 (4.69)	5.60 ± 0.49	0.90 ± 0.09
65 °C	707.43 ± 4.98	10.99 ± 0.09	62.01 ± 2.29	4.82 ± 0.21 (5.11)	5.87 ± 1.16	0.95 ± 0.23

**Table 4:** Electron and hole mobility values of PDTBSeVTT-2TF:PC_71_BM blend films processed at different temperatures.

Processing Temperatures (°C)	Blend e^−^ Mobility	Blend h^+^ Mobility	h^+^/e^−^ Ratio
	µ_e_ (cm^−2^·V^−1^·s^−1^)	µ_h_ (cm^−2^·V^−1^·s^−1^)	µ_e_/µ_h_
50	2.02 × 10^−4^	1.92 × 10^−4^	0.95
65	2.65 × 10^−4^	3.11 × 10^−4^	1.17

## Supplementary Materials

Supplementary materials are available online at www.mdpi.com/2073-4360/9/7/272/s1.
