# Supplementary material for: Novel Two-Dimensional Conjugated Polymer Containing Fluorinated Bithiophene as Donor and Benzoselenodiazole as Acceptor Units with Vinyl-Terthiophene Pendants for Polymer Photovoltaic Cells
Source: Polymers (Basel). 2017 Jul 7;9(7):272. doi: 10.3390/polym9070272 (PMC6431906; doi:10.3390/polym9070272)
Supplement: Supplementary file 1 [file polymers-09-00272-s001.pdf]

Supporting Information

Novel Two-dimensional Conjugated Polymer Containing Fluorinated Bithiophene as Donor and Benzoselenodiazole as Acceptor Units with Vinyl-Terthiophene Pendants for Polymer Photovoltaic Cells

Rathinam Raja[1,*], Shengkai Luo[1,3], Chuen-Yo Hsiow[1], Syang-Peng Rwei[3], Leeyih Wang[1,2,*]

[1]Center for Condensed Matter Sciences, National Taiwan University, Taipei, 10617, Taiwan
[2]Institute of Polymer Science and Engineering, National Taiwan University, Taipei, 10617, Taiwan
[3]Institute of Organic and Polymeric Materials, National Taipei University of Technology, Taipei, 10608, Taiwan

*Corresponding Author; E-mail: rajaorgchem80@gmail.com ; leewang@ntu.edu.tw

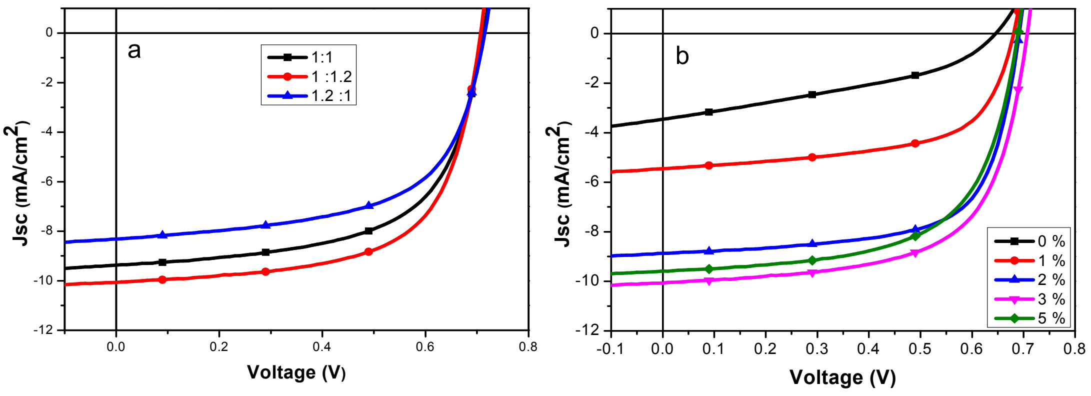

Figure S1. The current density-voltage (J-V) curves of PDTBSeVTT-2TF:$PC_{71}BM$ blend solution possessed at 50 °C (a) different weight ratio with 3 wt% of DIO additive, (b) weight ratio 1:1.2 with different wt% of DIO additive under the illumination of AM1.5G, 100 mW/cm^2.

Table S1. (**a**) The current density-voltage (J-V) curves of PDTBSeVTT-2TF:$PC_{71}BM$ blend solution possessed at 50 °C in different weight ratio with 3 wt% of DIO additive under the illumination of AM1.5G, 100 mW/cm^2.

D/A	V_{oc} (mV)	J_{sc} (mA/cm^2)	FF (%)	PCE (%)	R_s (Ωcm^2)	R_{sh} (Ωcm^2)
1:1	714.00±4.9	9.29±0.18	61.54±0.91	4.08±0.11	6.41±0.52	0.8±0.06
1:1.2	710.83±2.76	10.15±0.18	62.78±1.09	4.53±0.12	5.54±0.47	0.89±0.08
1.2:1	714.17±4.93	8.35±0.17	60.23±1.41	3.59±0.09	6.86±0.39	0.69±0.07

Table S2. (**a**) The current density-voltage (J-V) curves of PDTBSeVTT-2TF:$PC_{71}BM$ (1:1.2 Wt%) blend solution possessed at 50 °C with different weight wt% of DIO additive under the illumination of AM1.5G, 100 mW/cm^2.

DIO	V_{oc} (mV)	J_{sc} (mA/cm^2)	FF (%)	PCE (%)	R_s (Ωcm^2)	R_{sh} (Ωcm^2)
0 %	630±45.53	3.56±0.14	38.49±1.16	0.86±0.08	35.39±4.07	0.34±0.02
1 %	699.17±21.39	5.45±0.19	58.31±3.34	2.22±0.21	10.77±2.06	0.74±0.08
2 %	688.33±13.44	8.89±0.13	67.76±1.12	4.14±0.12	5.45±0.41	1.17±0.17
3 %	688.33±13.44	8.89±0.13	67.76±1.12	4.14±0.12	5.45±0.41	1.17±0.17
5 %	692.5±10.1	9.58±0.19	62.20±1.02	4.13±0.15	6.53±0.59	0.97±0.16

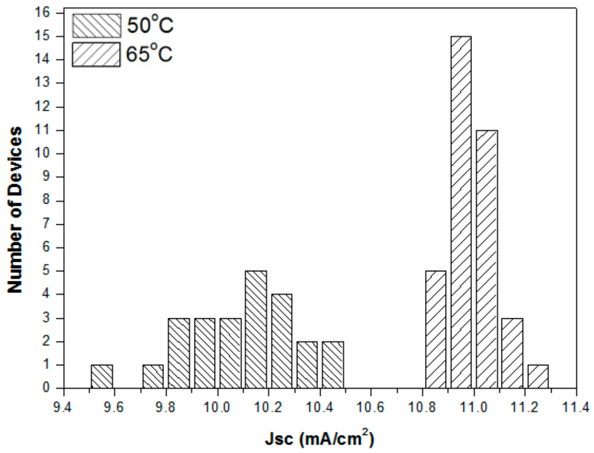

Figure S2 Statistical histogram chart of J_{SC} measured from 24 and 36 devices of PDTBSeVTT-2TF:$PC_{71}BM$ blend solutions processed at 50 °C and 65 °C, respectively.

.
